# Effects of Yeast Cell Wall Supplementation in Broiler Chickens Under Dietary Aflatoxin Challenge

**DOI:** 10.3390/vetsci13101061

**Published:** 2026-10-09

**Authors:** Melina Aparecida Bonato, Graziela Alves da Cunha Valini, Max Ingberman, Tadesse Mihret Yimam, Breno Castello Branco Beirão

**Affiliations:** 1 ICC Animal Nutrition, Jundiai 13211-223, Brazil; melina.bonato@iccbrazil.com.br; 2 Imunova Análise Biológicas, Curitiba 80215-182, Brazil; max@imunova.com.br (M.I.); breno.beirao@ufpr.br (B.C.B.B.); 3 Department of Basic Pathology, Universidade Federal do Paraná (UFPR), Curitiba 81531-980, Brazil; tadesse.mihret@ufpr.br

**Keywords:** poultry, aflatoxin, yeast cell wall, immune modulation, mycotoxin contamination

## Abstract

Aflatoxins (AFLAs) are highly prevalent mycotoxins in poultry production and are known to impair immune function and may reduce vaccine responsiveness. Therefore, this study evaluated whether dietary supplementation with yeast cell wall (YCW) could modulate the immune response under AFLA challenge and support vaccine response in broilers. A total of 144 one-day-old chicks were distributed into four treatments: negative control (NC), AFLA-contaminated diet (2.5 ppm), YCW (500 g/t), and YCW + AFLA. Birds were vaccinated against Newcastle Disease Virus (NDV) on D14. Body weight, hepatic pathology, intestinal permeability, circulating immune cell populations, phagocytic activity, and NDV-specific antibody titers were evaluated throughout the experimental period. AFLA exposure induced significant hepatic lesions and reduced body weight on D35 but did not alter the systemic immune parameters or intestinal permeability. Liver lesions were not prevented by the use of YCW, but body weight was positively correlated to the YCW intervention. Interestingly, YCW supplementation modulated immune responses in a context-dependent manner. In birds not exposed to AFLA, YCW increased anti-NDV antibody titers following vaccination. Contrarily, under AFLA challenge (and only in this situation), YCW supplementation was associated with changes in circulating immune cell populations and increased monocyte phagocytic activity, but in the presence of AFLA, YCW did not associate with increased anti-NDV titers. These findings indicate that YCW uniquely modulates selected innate and adaptive immune responses in broiler chickens exposed to AFLA, which is an important biological interaction with relevance to feed formulation for animal production.

## Simple Summary

Aflatoxins are toxic substances produced by fungi that frequently contaminate poultry feed, reducing bird performance and interfering with their immune system and vaccine responses. In this study, we tested whether ethanol yeast cell wall (YCW) could counteract the effects of aflatoxins. The effect of the toxin was restricted to liver lesions—which were not reversed by YCW supplementation—and to reduced body weight, which was rescued by the treatment. Interestingly, the YCW influenced immune responses differently depending on the presence of the aflatoxin challenge. Exclusively in unchallenged birds, supplementation increased antibody titers against Newcastle Disease Virus following vaccination. On the contrary, only under aflatoxin challenge was the YCW supplementation associated with changes in circulating immune cell populations and increased monocyte phagocytic activity. These findings suggest that ethanol yeast cell wall supplementation modulates immune responses in a context-dependent manner, with unique effects observed during aflatoxin challenge.

## 1. Introduction

Mycotoxins are natural contaminants of poultry feed and remain a persistent threat to broiler health and productivity. Among them, aflatoxin B1 (AFB1), produced primarily by *Aspergillus flavus* and *A. parasiticus*, is considered particularly relevant due to its high prevalence and toxicity in commercial production systems. In broiler chickens, aflatoxin (AFLA) can reduce body weight gain by up to 30% and impair immune responses [1,2]. Additionally, even at low concentrations, AFLA may impair immune function and reduce vaccination effectiveness in broilers [3]. These outcomes contribute to economic losses and increase the vulnerability of flocks to infectious diseases.

In this context, nutritional strategies that maintain immune competence and vaccination efficiency under mycotoxin exposure are of practical relevance. Yeast cell wall (YCW) products derived from *Saccharomyces cerevisiae* are widely recognized for their immunomodulatory properties, particularly due to their content of β-1,3/1,6-glucans and mannans [4,5]. These components interact with pattern-recognition receptors on immune cells, supporting phagocytic function, antigen presentation, and the development of effective adaptive responses. In addition, YCW can physically bind mycotoxins in the gastrointestinal tract, helping to reduce their absorption and subsequent systemic toxicity [6,7].

Previous studies in poultry have shown that YCW supplementation can mitigate the detrimental effects of mycotoxins, supporting performance and immune parameters under AFLA challenge [8,9,10]. We hypothesized that dietary supplementation with YCW derived from *Saccharomyces cerevisiae* would attenuate the negative impact of AFLA on the immune system of broilers and sustain the effectiveness of vaccination against Newcastle Disease Virus (NDV). The results showed interesting and previously unreported interactive effects between AFLA and YCW on broiler immunity.

## 2. Materials and Methods

A total of 144 one-day-old Cobb 500 broilers (mixed sexes) were housed in floor pens with wood-shaving litter under standard management conditions, with *ad libitum* access to feed and water. Stocking densities and environmental temperatures followed commercial conditions [11]. Diets were obtained from commercial suppliers and were provided in mash form and formulated according to bird age (pre-starter, starter and grower) to meet or exceed the nutritional requirements of broilers [12]. All procedures were approved by the Animal Experimentation Ethics Committee of Imunova Análises Biológicas (protocol CEUA 009/2020).

Birds were randomly assigned to four treatment groups, each containing 36 birds divided into four replicates of nine birds each: (1) negative control (NC, basal diet without aflatoxin or additive), (2) aflatoxins B1 and B2 (AFLA, basal diet + 2.5 ppm of total AFLA) [13], (3) yeast cell wall (YCW, basal diet + 500 g/metric ton of sugarcane YCW [ImmunoWall^®^, 30% β-glucans and 20% MOS, ICC, Brazil]), and (4) YCW + AFLA (basal diet + 500 g/metric ton of YCW + 2.5 ppm AFLA). AFLA contamination was achieved by incorporating naturally contaminated corn into the diet. The actual concentrations of aflatoxin B1 and B2 were confirmed by liquid chromatography–tandem mass spectrometry (Laboratory LAMIC, Brazil), averaging 2.24 ± 0.28 ppm of AFLA B1 and 133.77 ± 14.84 ppb of AFLA B2 (lower limit of detection = 0.5 ppb). Control feed without contamination had 1.67 ± 0.74 ppb AFLA B1 and undetectable levels of AFLA B2. Contaminated feed and/or YCW supplementation were provided throughout the experiment.

Body weights were monitored at each sample collection. All birds were vaccinated against Newcastle Disease Virus (NDV) on day 14 via ocular instillation with a live vaccine (Poulvac® NDW, Ulster 2C strain, Zoetis Inc., Parsippany, NJ, USA)). Blood samples were collected on days 14, 28, and 35 to determine NDV-specific antibody titers by ELISA (IDEXX^®^, Westbrook, ME, USA). All sampling on D14 occurred in animals that were not vaccinated and were culled and removed from the trial at that time point. Cell-mediated and innate immune responses were evaluated at multiple time points. On days 7, 14, 28 and 35, lymphocyte subpopulations were characterized by flow cytometry using chicken-specific monoclonal antibodies (Southern Biotech, Birmingham, AL, USA) [anti-CD45 (pan-leukocyte marker, clone LT-40), SPRD-coupled; anti-CD4 (clone CT-4), anti-CD8 alpha (CT-8) and anti-Kul-1 (Kul-01) were fluorescein isothiocyanate (FITC)-coupled; anti-TCR Vβ1 (TCR2), anti-CD28 (Av7), anti-Bu-1 (Av20) and anti-MHC II (2G11) were phycoerythrin (PE)-labelled]. Flow cytometry methods were conducted as previously described [14]. Phagocytic activity of heterophils and macrophages was assessed with pHrodo™ Green Zymosan Bioparticles (Thermo Fisher Scientific, Waltham, MA, USA). Intestinal permeability was determined on days 7, 14, 28 and 35 by oral pipetting of fluorescein isothiocyanate–dextran (FITC-dextran, 3–5 kDa, Sigma-Aldrich, St. Louis, MO, USA) at 1.1 mg/chick in 200 μL of PBS, followed by plasma sampling (blood in EDTA tubes) 2 h after administration for fluorescence measurement. Intestinal permeability was measured in 8 birds per group at each time point (*n* = 8). Protocols for phagocyte and intestinal permeability measurement were previously published by our group [15].

Livers were sampled in formalin for histopathological analysis on days 14 and 35. Lesions were scored as follows: Grade 1: Swelling with mild to moderate vacuolization or hydropic degeneration. Occasional mild necrosis and interstitial inflammatory cell infiltration by heterophils or by a combination of heterophils and mononuclear cells, in central or pericentral areas, along with mild hemorrhages and some mononuclear hyperplasia. Grade 2: Lesions as above, but more pronounced. More intense inflammatory response involving heterophils, often including immature segmented heterophilic cells and mononuclear cells with macrophages, accompanied by diffuse hemorrhages, frequently with sinusoidal collapse. Low frequency of portal fibrosis. Grade 3: Marked vacuolization and necrosis, with periportal fibrosis and periportal infiltration by mononuclear cells involving macrophages, in addition to multifocal bile duct hyperplasia. Portal or scattered fibrosis may occur, along with hemorrhages.

Data were tested for normality using the D’Agostino–Pearson test. Parametric data were analyzed by two-way ANOVA (time vs. treatment) followed by Tukey’s multiple comparison test, whereas nonparametric data were analyzed using the Kruskal–Wallis test. Statistical significance was set at *p* < 0.05. The *p*-values for significant sources of variation in two-way ANOVA are shown with each graph. All analyses were performed using GraphPad Prism (version 9, GraphPad Software, San Diego, CA, USA).

## 3. Results

### 3.1. Body Weight

Body weights were increased by the addition of YCW on D28 and D35. AFLA had reduced body weight on D35. On this date, YCW + AFLA was associated with increased body weight (Figure 1).

### 3.2. Hepatic and Intestinal Damage by Aflatoxin Challenge

AFLA exposure had no effect on intestinal permeability, as measured by FITC-dextran passage (Figure 2a), but induced significant liver damage (Figure 2b). Group AFLA showed significant lesions at both observation points, with a greater tendency toward chronification at D35, characterized by a low frequency of fibrosis and ductal reaction. Group YCW presented a low degree of injury, similar to group NC at D14. However, it exhibited swelling lesions at D35. Group YCW + AFLA, in turn, showed severe lesions of similar intensity on both collection days.

### 3.3. Antibody Response to NDV Vaccination

Supplementation with YCW increased anti-Newcastle Disease Virus antibody titers compared to all other groups at D35, following NDV vaccination at D14 (Figure 3).

### 3.4. Circulating Immunophenotype

The AFLA group did not have differences in the circulating immunophenotype in comparison to the NC group. AFLA challenge did not change any of the measured cell subsets in relation to the NC group, but the YCW + AFLA group had changes (in relation to NC) in CD4 and CD8 subsets before and on the day of NDV vaccination (D14), and also increased Kul^+^/MHCII^-^ cells and total leukocyte count after vaccination. Specifically, YCW + AFLA was different from NC on D7 in relation to CD8-CD28+, on D14, in relation to CD4^-^TCRV**β**1^+^, CD4^+^TCRV**β**1^+^, CD8^+^CD28^−^ and CD8^+^CD28^+^, and on D28 in relation to Kul^+^MHC II^−^ and total leukocyte counts (Table S1).

### 3.5. Phagocytic Activity

The phagocytic activity of heterophils was not altered by treatments (Figure 4a). YCW + AFLA supplementation increased monocyte phagocytic activity compared with all other groups at D35. When all dates were considered in a two-way ANOVA, YCW + AFLA showed higher monocyte phagocytic activity than AFLA (*p* = 0.0183) (Figure 4b).

## 4. Discussion

Aflatoxins are among the most relevant mycotoxins affecting poultry production, primarily due to their hepatotoxic effects and their potential to impair immune function. In the present study, dietary exposure to AFLA induced evident hepatic lesions, confirming the biological effectiveness of the challenge model. The liver was the main target tissue affected by AFLA exposure, which is consistent with its central role in aflatoxin metabolism and toxicity [16]. Birds also showed reduced weight by the end of the trial in the presence of aflatoxin. After intestinal absorption, AFLA is metabolized in the liver into reactive metabolites that can induce hepatocellular damage and may interfere with protein synthesis [7,17,18,19]. In our trial, the effects of AFLA on the liver could not be overcome by YCW treatment, but body weights were higher in AFLA-challenged birds supplemented with YCW.

Under the conditions of this study, AFLA challenge induced hepatic injury without producing broad detectable changes in intestinal permeability or peripheral immune cell subsets. The AFB1 dose used here (~2.2 ppm) was intentionally high compared to levels currently observed in most commercial operations [20,21], but was selected to evaluate the effects of YCW under a robust aflatoxin challenge. This concentration was used to ensure experimentally induced disease, since clinically evident aflatoxicosis is often aggravated by comorbidities that were intentionally absent from this experimental model [22]. AFLA dose was calculated based on aflatoxin B1, the main toxic variant. Since contamination was derived from naturally contaminated corn, B2 was present and measured for completeness, but was not controlled. Co-contamination with these aflatoxin variants can enhance immune suppression [23]. The dose was not intended to induce acute aflatoxicosis, but rather to produce sustained subclinical-to-moderate toxic effects throughout the production cycle, allowing the evaluation of YCW under continuous aflatoxin exposure. Concentrations above 5 ppm are generally necessary for more severe acute clinical manifestations of aflatoxicosis.

The expected effects of AFLA B1 on intestinal integrity are much milder, even at high doses. Our findings are consistent with previous reports indicating that dietary AFLA at approximately 2 ppm may not significantly alter FITC-dextran intestinal permeability in poultry [24].

Previous studies have reported that aflatoxins and other mycotoxins may impair humoral immunity by reducing vaccine-induced antibody titers, including responses to NDV [2,3,25]. However, unlike hepatic effects, immune alterations induced by aflatoxins are less predictable and depend on multiple experimental variables, including dose, duration of exposure, bird age, immune status, and the immune marker being evaluated [26,27,28,29].

Nevertheless, our data on immunity were interesting and suggest interactions occurring between YCW and AFLA. We anticipated that YCW would counteract AFLA since the cell wall is itself immunomodulatory, but also because it can capture the mycotoxin via various possible chemical interactions between them [30]. However, their effects on immunity were not linearly additive, indicating more complex interactions between YCW and AFLA or between each of them and immune cells.

Anti-NDV antibody titers at day 35 showed predictable results regarding the role of YCW, indicating a positive effect on vaccine responsiveness in non-challenged birds. This response is expected and is associated with the presence of β-glucans and mannans in YCW, which are recognized as immunomodulatory compounds. β-glucans, particularly β-1,3/1,6-glucans, can be detected by pattern-recognition receptors expressed on phagocytic and antigen-presenting cells, triggering intracellular signaling pathways that may promote cytokine-mediated communication and contribute to the coordination between innate and adaptive immune responses [31].

Increased immune activation may be one of the effects of YCW observed here: YCW by itself induced liver swelling at D35. There are reports of changes in liver function and markers of liver health in ruminants treated with YCW, and this has been speculated to be correlated with increased tissue inflammatory activity due to the treatment [32,33]. Immune activation induced by YCW at D35 thus may be responsible for both increased anti-NDV titers and liver inflammatory lesions, although this is not a common finding in the literature of poultry science.

Interestingly, the immune effects of YCW were not fully replicated under AFLA challenge. AFLA alone did not significantly reduce anti-NDV antibody titers compared with the NC group, but the YCW + AFLA group did not reach the antibody response observed in birds supplemented with YCW alone. Thus, under the conditions of this study, the effect of YCW on humoral vaccine response was most evident in the absence of AFLA challenge. If the results of AFLA + YCW were additive, it would be expected that YCW would have induced higher antibody titers in the presence of the toxin, since AFLA was not deleterious to the antibody response.

The interaction between YCW and AFLA became more evident in the circulating immune cell repertoire. Although YCW and AFLA alone did not alter circulating immunity, the effect of YCW became evident under AFLA challenge in peripheral immune cell subsets and innate immune function. Birds receiving YCW + AFLA showed changes in circulating immune cell populations, including alterations in T-cell subsets before and at the time of NDV vaccination (D14), followed by changes in Kul^+^MHC II^-^ and total leukocyte counts after vaccination. The overall changes in the phenotype do not amount to a clearly defined immunological outcome, such as immune activation or suppression.

Therefore, there was an unexpected interaction between YCW and AFLA, whose mechanism is unknown. One possibility (among many others) is that the physical binding between aflatoxins and glucans and mannans in YCW changed how these wall molecules interact with immune receptors. While glucans and mannans are immunomodulatory, their capacity to bind to immune receptors is dependent on the conformation of these molecules [34]. Aflatoxin physically interacts with glucans and mannans [35], and it is possible that changes in the conformation of the YCW also alter how wall molecules interact with immune receptors such as dectin, thus explaining the results seen here.

The functional response of innate immune cells further supports the interpretation of an interaction between YCW and AFLA. Monocyte phagocytic activity was increased in the YCW + AFLA group on day 35 and when all sampling dates were considered together. YCW + AFLA showed higher monocyte phagocytic activity than AFLA alone, suggesting that YCW supplementation may have supported monocyte/macrophage functional activity under aflatoxin exposure, but not when YCW was administered alone. However, this response appeared to be cell-type specific, since it was not observed in heterophils, which also express a putative dectin receptor for mannans and glucans [36]. With our data, it is not possible to speculate on the different responses between monocytic cells and heterophils to YCW + AFLA. Limitations of the study include the limited effects of AFLA on immune suppression. This hampers comparison to field data, where this concentration of the toxin is usually associated with immune dysfunction. In addition, only a single AFLA concentration (2.5 ppm total AFLA, with approximately 2.24 ppm AFB1) and a single YCW inclusion level were tested, so dose-dependent effects of either could not be evaluated, and the findings may not extend to lower or higher exposures or to other exposure durations. Contamination came from naturally contaminated corn, so AFB2 (approximately 134 ppb) was present but not controlled; its effects cannot be separated from those of AFB1, and the contribution of other naturally co-occurring contaminants cannot be excluded. Intestinal permeability was assessed only by FITC-dextran passage at a single time after administration. Finally, the study was conducted under controlled conditions without concurrent comorbidities, with one YCW product and one vaccine (NDV), so extrapolation to field conditions should be made with caution.

## 5. Conclusions

Supplementation with sugarcane yeast cell wall (YCW) modulates immune responses in broiler chickens in a context-dependent manner. In unchallenged birds, YCW supplementation enhances anti-NDV antibody titers, whereas under AFLA challenge, it modulates circulating immune cell populations and increases monocyte phagocytic activity. Limitations, including the use of a single aflatoxin dose and the limited immunosuppressive effect of AFLA, are discussed above and should be considered when extrapolating to field conditions.

## Figures and Tables

**Figure 1:**
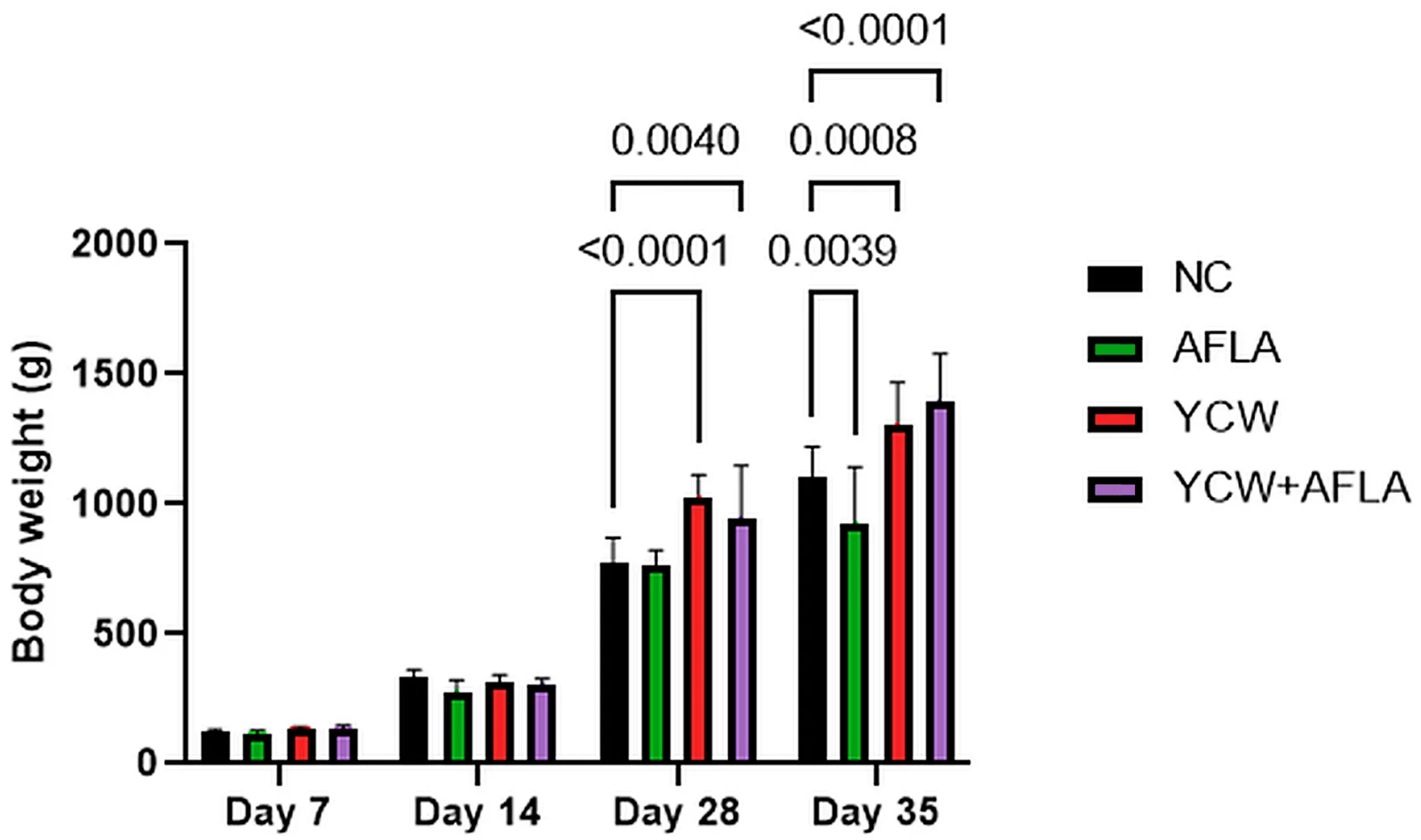
Body weight was increased by YCW treatment and decreased by AFLA challenge. Bars show mean and standard deviation. Multiple comparison test comparing groups to NC. Two-way ANOVA indicated “Dates” and “Groups” as sources of variation, with significant interactions between them (*p* < 0.0001 for the two factors and interaction). *n* = 8 birds/group at each time point.

**Figure 2:**
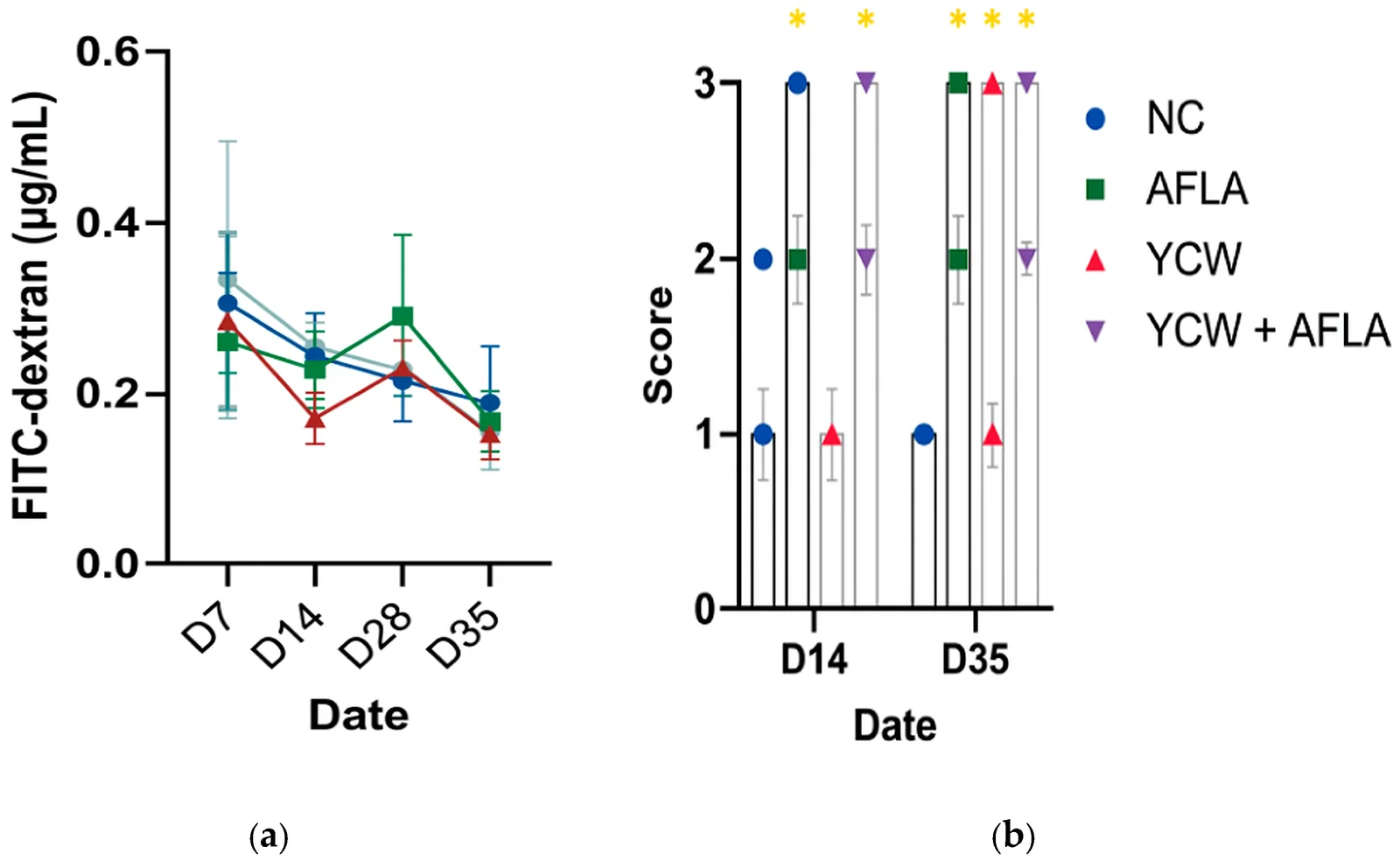
Aflatoxin induced liver lesions but did not alter intestinal permeability. (**a**) Intestinal permeability assessed by FITC-dextran passage, showing means and standard deviations. Two-way ANOVA indicated “Date” (*p* < 0.0001) as the source of variation. (**b**) Liver lesion scoring, showing individual values and group median. Significant differences against NC are shown by asterisks (* *p* < 0.05; *** *p* < 0.001). *n* = 8 birds/group at each time point.

**Figure 3:**
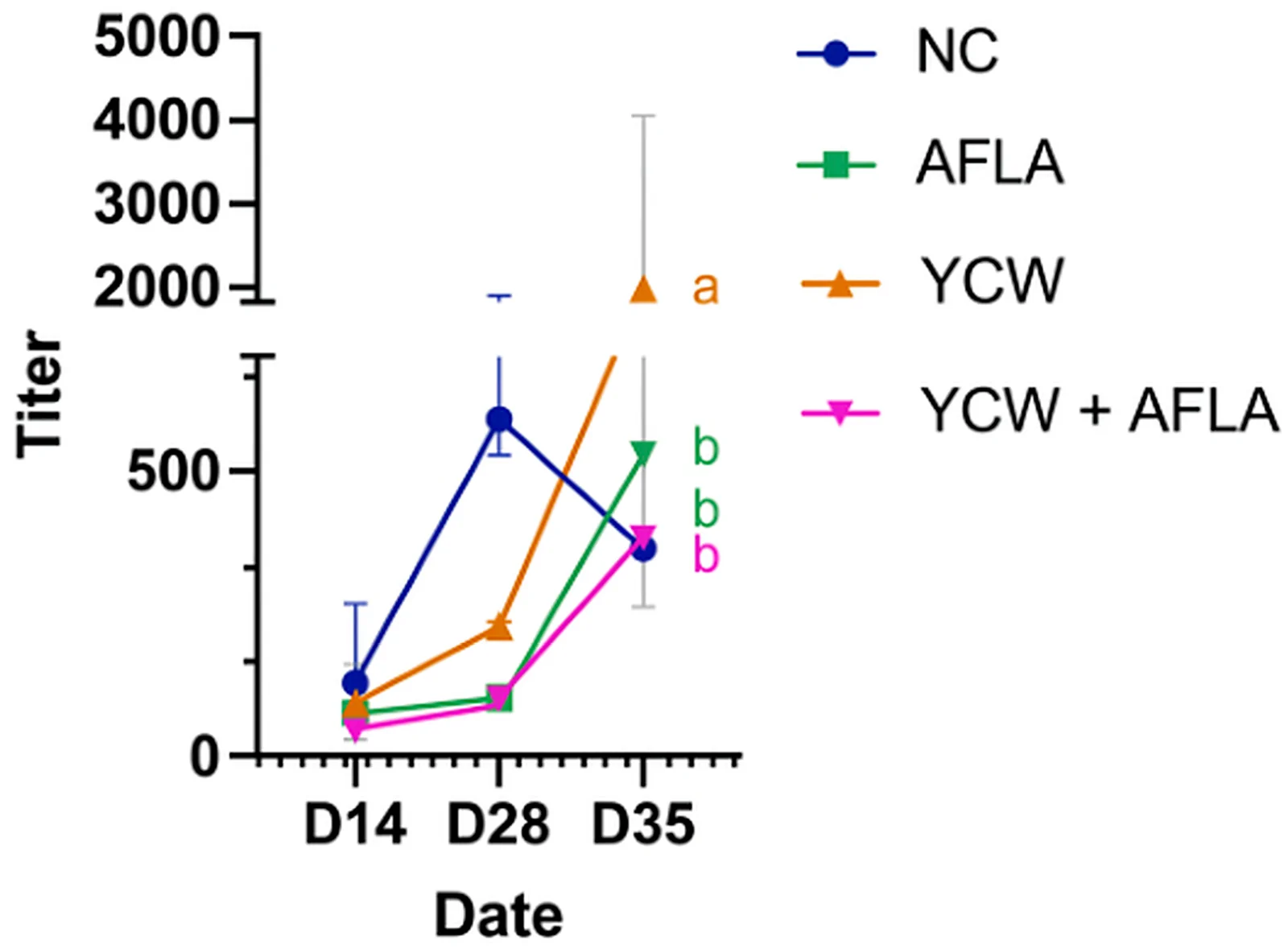
Yeast cell wall treatment (YCW) increased anti-Newcastle Disease Virus (NDV) titer. ELISA for NDV antibody titration. The graph shows the mean and standard deviation. Statistically different treatments on a given date are indicated by varying letters next to the respective date. Two-way ANOVA indicated “Date” (*p* = 0.0012) as a source of variation, and an interaction between date and treatments (*p* = 0.0152). *n* = 8 birds/group at each time point.

**Figure 4:**
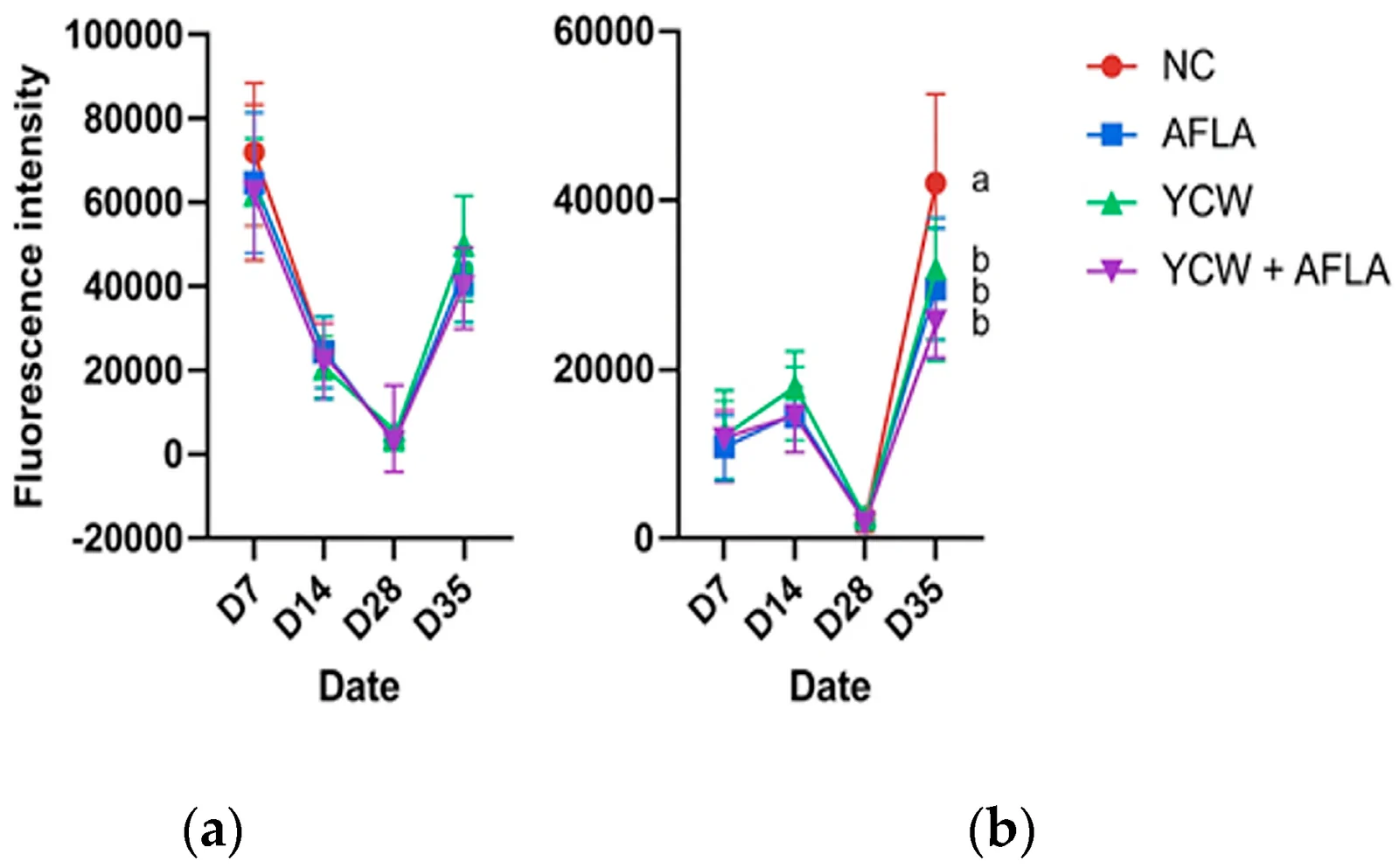
Aflatoxin challenge with yeast cell wall treatment (YCW + AFLA) led to higher monocyte phagocytic activity. Phagocytic activity was measured by fluorescence intensity in flow cytometry. The graph shows the mean and standard deviation. (**a**) Heterophil phagocytic activity. Two-way ANOVA indicated “Date” (*p* < 0.0001) as the source of variation, with no interaction between date and treatments. (**b**) Monocyte phagocytic activity. Statistically different groups on a given date are indicated by different letters. Two-way ANOVA indicated “Date” (*p* < 0.0001) and “Treatment” (*p* = 0.021) as sources of variation, and an interaction between date and treatment (*p* = 0.032). *n* = 8 birds/group at each time point.

## Data Availability

The original contributions presented in this study are included in the article. Further inquiries can be directed to the corresponding author.

## Supplementary Materials

The following supporting information can be downloaded at: https://www.mdpi.com/article/10.3390/vetsci13101061/s1. Table S1: Peripheral blood immunophenotype of broilers challenged with or without aflatoxin (AFLA) and with/without supplementation of sugarcane yeast cell wall (YCW) for the entire trial. NDV vaccination was administered on day 14.

## Abbreviations

The following abbreviations are used in this manuscript:

AFLA	Aflatoxin
NC	Negative control
YCW	Yeast cell wall
NDV	Newcastle Disease Virus

## References

[1] Rawal S., Kim J.E., Coulombe R. (2010). Aflatoxin B1 in poultry: Toxicology, metabolism and prevention. Res. Vet. Sci..

[2] Nemati Z., Karimi A., Besharati M. Impact of aflatoxin contaminated feed and yeast cell wall supplementation on immune system in broiler chickens. Proceedings of the International Conference on Innovations in Chemical & Agricultural Engineering.

[3] Hou L., Qiu H., Li A., Dong J., Zhu L., Liu G., Chen F. (2022). Effects of aflatoxin B1 on growth performance, antioxidant status, immune response, and pro-inflammatory cytokine mRNA expression in ISA chicks. Front. Vet. Sci..

[4] Morales-López R., Auclair E., García F., Esteve-Garcia E., Brufau J. (2009). Use of yeast cell walls; beta-1, 3/1, 6-glucans; and mannoproteins in broiler chicken diets. Poult. Sci..

[5] Zhang B., Guo Y., Wang Z. (2008). The modulating effect of 棺-1, 3/1, 6-glucan supplementation in the diet on performance and immunological responses of broiler chickens. Asian-Australas. J. Anim. Sci..

[6] Weaver A.C., Weaver D.M., Adams N., Yiannikouris A. (2024). Meta-analysis of the effects of yeast cell wall extract supple-mentation during mycotoxin challenges on the performance of laying hens. Toxins.

[7] Xu R., Yiannikouris A., Shandilya U.K., Karrow N.A. (2023). Comparative assessment of different yeast cell wall-based mycotoxin adsorbents using a model-and bioassay-based in vitro approach. Toxins.

[8] Mendieta C.R., Gómez G.V., Del Río J.C.G., Cuevas A.C., Arce J.M., Ávila E.G. (2018). Effect of the Addition of Saccharomyces Cerevisiae Yeast Cell Walls to Diets with Mycotoxins on the Performance and Immune Responses of Broilers. J. Poult. Sci..

[9] Liu N., Wang J.Q., Jia S.C., Chen Y.K., Wang J.P. (2018). Effect of yeast cell wall on the growth performance and gut health of broilers challenged with aflatoxin B1 and necrotic enteritis. Poult. Sci..

[10] Hernández-Ramírez J.O., Merino-Guzmán R., Téllez-Isaías G., Vázquez-Durán A., Méndez-Albores A. (2021). Mitigation of AFB1-Related Toxic Damage to the Intestinal Epithelium in Broiler Chickens Consumed a Yeast Cell Wall Fraction. Front. Vet. Sci..

[11] Cobb-Vantress (2021). Cobb Broiler Management Guide.

[12] Albino L., Donzele J., Gomes P., Oliveira R., Lopes D., Ferreira A., Barreto S., Euclides R. (2017). Tabelas Brasileiras para Aves e Suínos: Composição de Alimentos e Exigências Nutricionais.

[13] Huff W.E., Kubena L.F., Harvey R.B., Corrier D.E., Mollenhauer H.H. (1986). Progression of Aflatoxicosis in Broiler Chickens. Poult. Sci..

[14] Beirão B., Ingberman M., Fávaro C., Caron L. (2020). Reference parameters of peripheral immune cell populations in broiler chickens using a large database. Br. Poult. Sci..

[15] Bonato M., Borges L.L., Ingberman M., Fávaro C., Mesa D., Caron L.F., Beirão B.C.B. (2020). Effects of yeast cell wall on immunity, microbiota, and intestinal integrity of Salmonella-infected broilers. J. Appl. Poult. Res..

[16] Wang Y., Wang X., Li Q. (2023). Aflatoxin B1 in poultry liver: Toxic mechanism. Toxicon.

[17] Benkerroum N. (2020). Chronic and acute toxicities of aflatoxins: Mechanisms of action. Int. J. Environ. Res. Public Health.

[18] Turner P.C. (2013). The molecular epidemiology of chronic aflatoxin driven impaired child growth. Scientifica.

[19] Williams J.H., Phillips T.D., Jolly P.E., Stiles J.K., Jolly C.M., Aggarwal D. (2004). Human aflatoxicosis in developing countries: A review of toxicology, exposure, potential health consequences, and interventions. Am. J. Clin. Nutr..

[20] Naveed M., Haleem K.S., Ghazanfar S., Tauseef I., Bano N., Adetunji C.O., Saleem M.H., Alshaya H., Paray B.A. (2022). Quantitative Estimation of Aflatoxin Level in Poultry Feed in Selected Poultry Farms. BioMed Res. Int..

[21] Kobashigawa E., Corassin C.H., Franco L.T., Uliana R.D., Oliveira C.A.F.d. (2019). Aflatoxins and fumonisins in feed from a broiler operation system from São Paulo state, Brazil. Ciênc. Rural.

[22] Huff W.E., Kubena L.F., Harvey R.B., Doerr J.A. (1988). Mycotoxin interactions in poultry and swine. J. Anim. Sci..

[23] Giambrone J.J., Diener U.L., Davis N.D., Panangala V.S., Hoerr F.J. (1985). Effects of purified aflatoxin on broiler chickens. Poult. Sci..

[24] Galarza-Seeber R., Latorre J.D., Bielke L.R., Kuttappan V.A., Wolfenden A.D., Hernandez-Velasco X., Merino-Guzman R., Vicente J.L., Donoghue A., Cross D. (2016). Leaky gut and mycotoxins: Aflatoxin B1 does not increase gut permeability in broiler chickens. Front. Vet. Sci..

[25] Ortatatli M., Oğuz H., Hatipoğlu F., Karaman M. (2005). Evaluation of pathological changes in broilers during chronic aflatoxin (50 and 100 ppb) and clinoptilolite exposure. Res. Vet. Sci..

[26] Olariu R.M., Fiţ N.I., Bouari C.M., Nadăş G.C. (2025). Mycotoxins in broiler production: Impacts on growth, immunity, vaccine efficacy, and food safety. Toxins.

[27] Yunus A.W., Razzazi-Fazeli E., Bohm J. (2011). Aflatoxin B1 in affecting broiler’s performance, immunity, and gastrointestinal tract: A review of history and contemporary issues. Toxins.

[28] Peng X., Bai S., Ding X., Zeng Q., Zhang K., Fang J. (2015). Pathological changes in the immune organs of broiler chickens fed on corn naturally contaminated with aflatoxins B1 and B2. Avian Pathol..

[29] Tessari E.N.C., Oliveira C.A.F., Cardoso A.L.S.P., Ledoux D.R., Rottinghaus G.E. (2006). Effects of aflatoxin B1 and fumonisin B1 on body weight, antibody titres and histology of broiler chicks. Br. Poult. Sci..

[30] de Lima Schlösser L.M., Simões C.T., Sarturi J.A., da Silva C.R., Laber I.F., Franco D.S.P., Mallmann C.A. (2024). Adsorption of aflatoxin B1 by different antimycotoxin additives: Bentonite, clinoptilolite, and beta-glucans extracted from yeast cell wall. Mycotoxin Res..

[31] Petravić-Tominac V., Zechner-Krpan V., Grba S., Srečec S., Panjkota-Krbavčić I., Vidović L. (2010). Biological effects of yeast β-glucans. Agric. Conspec. Sci..

[32] Carpinelli N.A., Halfen J., Michelotti T.C., Rosa F., Trevisi E., Chapman J.D., Sharman E.S., Osorio J.S. (2023). Yeast Culture Supplementation Effects on Systemic and Polymorphonuclear Leukocytes’ mRNA Biomarkers of Inflammation and Liver Function in Peripartal Dairy Cows. Animals.

[33] Han X., Liu T., Zeng H., Liu J., Lu Z., Xu J., Wang J., Yang X., Zhu S., Ran H. (2026). Supplemental feeding of yeast cell wall promotes growth of Tibetan sheep by altering rumen fermentation and improving rumen microbiota and liver metabolism. BMC Microbiol..

[34] Huo R., Wuhanqimuge, Zhang M., Sun M., Miao Y. (2025). Molecular dynamics modeling of different conformations of beta-glucan, molecular docking with dectin-1, and the effects on macrophages. Int. J. Biol. Macromol..

[35] Vendele I., Willment J.A., Silva L.M., Palma A.S., Chai W., Liu Y., Feizi T., Spyrou M., Stappers M.H., Brown G.D. (2020). Mannan detecting C-type lectin receptor probes recognise immune epitopes with diverse chemical, spatial and phylogenetic heterogeneity in fungal cell walls. PLoS Pathog..

[36] Nerren J.R., Kogut M.H. (2009). The selective Dectin-1 agonist, curdlan, induces an oxidative burst response in chicken heterophils and peripheral blood mononuclear cells. Vet. Immunol. Immunopathol..

