# Supplementary material for: Effects of Yeast Cell Wall Supplementation in Broiler Chickens Under Dietary Aflatoxin Challenge
Source: Vet Sci. 2026 Oct 9;13(10):1061. doi: 10.3390/vetsci13101061 (PMC13709935; doi:10.3390/vetsci13101061)
Supplement: Supplementary file 1 [file vetsci-13-01061-s001.zip › vetsci-4543478-supplementary.pdf]

Table S1. Peripheral blood immunophenotype of broilers challenged with or without Aflatoxin (AFLA) and with/without supplementation of sugarcane yeast cell wall (YCW) for the entire trial. NDV vaccination was administered on day 14.

Date	Group		CD4^{+} TCRVβ1^{-}	CD4^{-} TCRVβ1^{+}	CD4^{+} TCRVβ1^{+}	CD8^{+} CD28^{-}	CD8^{-} CD28^{+}	CD8^{+} CD28^{+}	Kul^{+} MHC II^{-}	Kul^{-} MHCII^{+}	Kul^{+} MHCII^{+}	Bu-1^{+}	Total events	MHC II intensity Kul+	MHC II intensity Kul-	CD4/CD8
D7	NC	Mean	9.22	4.60	32.22	2.71	25.05 b	11.97	7.72	4.28	9.80	12.29	17262	43998	53921	2.91
		SD	3.45	2.19	7.95	1.59	7.69	3.10	3.75	1.34	4.01	3.43	11262	10014	20920	0.85
	AFLA	Mean	8.67	8.73	32.49	3.32	29.08 ab	13.47	7.44	7.43	13.17	14.91	14008	30063	45277	2.49
		SD	2.16	6.53	6.99	1.21	6.12	3.21	3.15	5.30	11.59	3.23	2942	9060	16864	0.55
	YCW	Mean	9.45	5.94	28.85	3.45	27.63 ab	12.54	6.08	3.49	8.96	13.00	15603	44043	62685	2.64
		SD	2.57	2.59	6.73	1.58	9.05	3.35	2.01	0.83	4.22	4.15	2136	22171	20115	1.22
	YCW + AFLA	Mean	9.60	5.99	35.22	2.15	35.00 a	11.68	6.33	4.22	11.87	15.51	18025	33450	50190	3.79
		SD	3.40	2.39	8.91	1.61	8.43	5.00	2.91	2.94	5.26	4.01	4463	11001	18078	1.82
D14	NC	Mean	5.86	8.52 a	19.32 a	4.98 a	23.90	15.70 b	4.87	8.36	6.34	0.04	16666	98006 ab	100681	1.36
		SD	2.27	2.84	5.55	3.28	7.78	5.48	1.74	4.92	2.80	0.05	6768	11860	34311	0.60
	AFLA	Mean	5.39	6.71 ab	16.55 ab	2.27 ab	19.45	18.20 ab	5.26	6.28	7.57	0.02	21191	104977 a	109034	1.13
		SD	2.13	1.68	6.00	1.43	6.91	7.46	1.03	1.73	3.25	0.03	6684	25929	27133	0.28
	YCW	Mean	5.66	7.57 a	19.21 a	3.00 ab	23.30	21.06 a	5.94	8.27	8.52	0.03	16902	95201 ab	104325	1.14
		SD	1.90	1.97	4.50	1.39	7.62	6.72	2.12	3.11	2.38	0.03	5249	24029	25723	0.46
	YCW + AFLA	Mean	2.95	6.01 b	11.77 b	1.47 b	16.32	10.35 c	3.88	4.09	3.48	0.02	36777	83802 b	116565	1.29
		SD	1.24	1.78	4.13	0.68	5.40	3.75	1.71	1.69	1.37	0.03	14388.	14517	12503	0.37
D28	NC	Mean	1.97	12.44	9.14	2.12	9.53	3.73	2.67 b	1.47	8.54	4.47 ab	53170b	17586	34390	2.02
		SD	1.16	8.98	2.78	1.00	8.27	1.47	0.96	0.70	2.92	9.32	21798	7579	11809	0.55
	AFLA	Mean	2.04	8.12	8.37	2.02	6.91	2.93	4.50 ab	3.94	10.89	1.63 b	55245 b	23582	40984	2.46
		SD	0.90	5.93	2.21	1.16	3.34	1.17	1.89	6.18	7.20	2.91	32936	12852	15416	0.99
	YCW	Mean	3.17	5.44	7.84	1.77	6.02	2.57	2.44 b	1.13	5.17	0.43 b	49196 b	13271	29425	2.57
		SD	2.56	1.92	2.64	0.48	1.84	0.42	0.96	0.30	2.68	0.18	22982	3920	11528	0.80
	YCW + AFLA	Mean	2.75	13.45	10.74	2.52	10.98	5.38	6.32 a	2.37	6.06	9.96 a	82642 a	15130	32129	2.03
		SD	2.30	7.88	3.79	2.29	3.71	2.18	4.37	0.69	2.69	8.02	21864	6977	15533	1.10
D35	NC	Mean	4.16	11.98	12.19	1.51	18.61	2.92	1.81	10.60 ab	21.67	5.43	52635	26464	28594	4.40
		SD	2.33	7.17	4.05	1.18	6.40	1.49	1.46	9.85	9.41	8.92	23940	11020	9657	2.18
	AFLA	Mean	3.98	11.57	11.94	1.54	15.95	4.59	1.62	17.75 a	23.23	5.00	41592	20538	21192	3.42
		SD	1.92	5.83	4.49	0.89	6.46	1.90	1.07	19.37	5.86	4.46	27850	6761	8032	2.36
	YCW	Mean	4.19	7.16	13.03	1.11	13.31	3.46	1.14	9.09 b	20.66	0.93	30843	18992	21548	4.25
		SD	1.37	1.89	5.61	0.69	6.11	1.80	0.57	4.66	9.56	0.54	8957	5178	7844	1.73
	YCW + AFLA	Mean	5.46	7.86	16.50	2.62	12.16	4.13	1.64	5.14 b	18.17	1.53	36671	34613	37111	3.58
		SD	1.21	2.10	3.00	1.27	3.57	2.20	0.45	1.79	7.30	1.26	10643	13838	15289	1.11
	Interaction		0.2679	0.0531	0.0513	0.0043	0.0208	0.0014	0.0374	0.3034	0.7961	0.0248	0	0	0	0.5339
	Date		<0.0001	0.0099	<0.0001	0.0045	<0.0001	<0.0001	<0.0001	<0.0001	<0.0001	<0.0001	<0	<0	<0	<0.0001

Group	0.7734	0.1294	0.7382	0.39	0.7553	0.1307	0.5342	**0.0319**	0.1039	0.0721	0	0	0	0.7679

Cell subpopulations are indicated as a % of total $CD45^+$. Differences between treatment groups on a specific date are indicated by different letters next to the mean values.

Abbreviations – AFLA = aflatoxin, YCW = Yeast cell wall, SD standard deviation, NC = negative control, D7-D35 = experimental days, Marker$^+$ = cells that are positive for the expression of the given marker; Marker$^-$ = cells that are negative for the expression of the given marker. “MHC II intensity” = expression levels of this receptor in the given Kul-01 population, which are indicated by fluorescence intensity. “CD4/CD8” is the ratio between these cell subtypes.
